# A biophysiological framework exploring factors affecting speech and swallowing in clinical populations: focus on individuals with Down syndrome

**DOI:** 10.3389/fpsyg.2023.1085779

**Published:** 2023-06-21

**Authors:** Aarthi Madhavan, Larissa Lam, Nicole M. Etter, Krista M. Wilkinson

**Affiliations:** Department of Communication Sciences and Disorders, The Pennsylvania State University, University Park, PA, United States

**Keywords:** speech, swallowing, sensorimotor control, Down syndrome, biophysiological framework

## Abstract

Speech and swallowing are complex sensorimotor behaviors accomplished using shared vocal tract anatomy. Efficient swallowing and accurate speech require a coordinated interplay between multiple streams of sensory feedback and skilled motor behaviors. Due to the shared anatomy, speech and swallowing are often both impacted in individuals with various neurogenic and developmental diseases, disorders, or injuries. In this review paper, we present an integrated biophysiological framework for modeling how sensory and motor changes alter functional oropharyngeal behaviors of speech and swallowing, as well as the potential downstream effects to the related areas of language and literacy. We discuss this framework with specific reference to individuals with Down syndrome (DS). Individuals with DS experience known craniofacial anomalies that impact their oropharyngeal somatosensation and skilled motor output for functional oral-pharyngeal activities such as speech and swallowing. Given the increased risk of dysphagia and “silent” aspiration in individuals with DS, it is likely somatosensory deficits are present as well. The purpose of this paper is to review the functional impact of structural and sensory alterations on skilled orofacial behaviors in DS as well as related skills in language and literacy development. We briefly discuss how the basis of this framework can be used to direct future research studies in swallowing, speech, and language and be applied to other clinical populations.

## Introduction

This article proposes a multidimensional theoretical framework for understanding how characteristics associated with the phenotype of Down syndrome (DS) may influence performance of swallow behavior and production of intelligible speech, as well as impacting language development and foundational literacy outcomes such as phonemic awareness and phoneme-grapheme correspondence. This framework takes as its starting point a model introduced by the first and third authors that considered food selection, swallow, and speech in healthy older individuals (Etter and Madhavan, 2020). This model was developed because of the unique needs in healthy older adults. One example is that older adults without a diagnosis related to dysphagia typically do not report swallowing changes, and instead make self-determined compensations like changing their diet or avoiding foods, which may be the result of early dysphagia and lead to negative consequences (Roy et al., 2007; Madhavan, 2020). These needs are often missed in models that are applicable to clinical populations.

**FIGURE 1:**
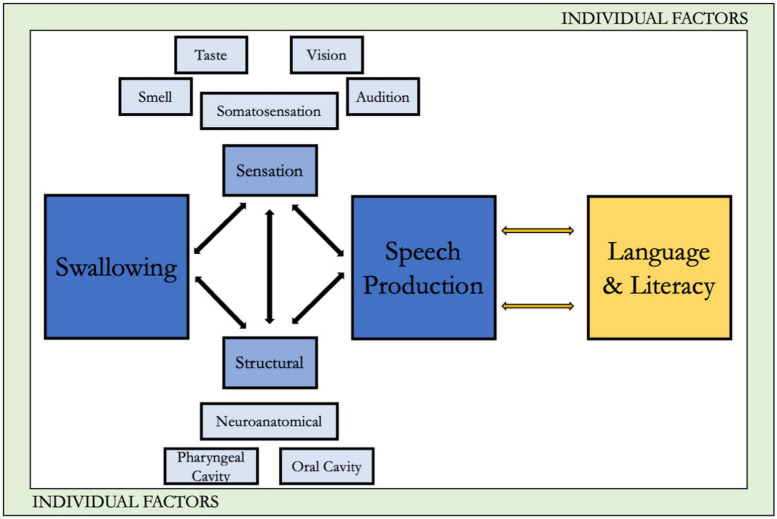
A biophysiologically-integrated framework of swallow, speech, and language/literacy.

We chose to expand this integrated framework to the DS population because despite our knowledge of heightened swallowing, speech, language, and literacy problems in individuals with DS, it has not consistently translated to improved clinical outcomes. This may be because management approaches used in DS often “borrow” techniques from other populations (Neil and Jones, 2018), however, these other populations do not have the syndrome-specific structural, functional, or physiologic dysmorphologies characteristic in DS. To improve precision interventions in DS, an integrated understanding of the unique phenotypical characteristics is an important early step. The framework is also consistent with the World Health Organization’s International Classification of Functioning (Centers for Disease Control [CDC], n.d.), with a particular focus on the effects of the characteristics associated with DS on body functions and structures and, in turn, activities and participation. Although we acknowledge the critical role of environmental factors as well, for the sake of space we focus primarily on the body functions and structures within DS. Moreover, our approach is consistent with recommendations for critical directions in DS research as suggested by an expert panel (Hendrix et al., 2020). Our extensions in DS include: (1) application of the framework within the context of the distinctive phenotypically linked structural and functional characteristics associated with DS, and (2) consideration of the potential downstream impacts of the phenotypic oropharyngeal characteristics in DS on language and early literacy skills, thus extending the analysis beyond swallowing to speech and related linguistic functions. Figure 1 presents the framework, with the original conceptualization along with the extension to language and literacy, as it relates to DS.

## Phenotypic characteristics in DS of relevance for the framework

In the United States, it is estimated that approximately 8 per 10,000 individuals are living with DS, a genetic disorder that results from a full or partial extra copy of chromosome 21 (Presson et al., 2013). Difficulties with speech intelligibility, swallowing, and language and literacy are reported throughout the lifespan in DS and are likely in part due to phenotypically linked structural, linguistic, and cognitive characteristics (Bruni et al., 2010). For instance, phenotypical differences in the structure and function of oral-motor mechanisms and in measures of cognition and language are well-documented in individuals with DS. An example of a structural change includes craniofacial anomalies that may cause obstruction in the airway at multiple levels in the respiratory system (Shott and Donnelly, 2004). These may impact functional speech production by affecting the motor processes involved in speech kinematics, in turn affecting speech intelligibility.

In addition to impacts on swallow and speech, the phenotypic characteristics in DS likely also impact both language and literacy outcomes across the lifespan. As we will outline in the second half of the paper, functions of language and literacy may also be affected both by difficulties in producing intelligible speech as well as potentially in hearing or perceiving spoken input. Although language and literacy learning are often considered to occur primarily in early to middle childhood, in reality these are lifelong learning activities, particularly for individuals with DS. For instance, Chapman et al. (2002) demonstrated through growth curve modeling that although the speed of growth changes, there is continued growth in both expressive and receptive syntax throughout adolescence in DS (Chapman et al., 2002). As Abbeduto and Thurman (2022), p. 1583 point out, literacy instruction often receives less attention for “students with intellectual disabilities as they get closer to exiting formal schooling and transitioning to adulthood, despite the reality that independence in adulthood depends critically on language and literacy.” However, the new motivations introduced by access to social media and for vocational skills in adolescence and adulthood mean that literacy too should continue to evolve across the lifespan, a point raised also raised by Abbeduto et al. (2007). Adding a further layer of complexity, individuals with DS demonstrate accelerated aging, especially in the brain (Lott and Head, 2005). Accelerated aging in the brain is thought to be as significant as 11 years earlier (Horvath et al., 2015), indicating that even though learning of academic skills continues into the third decade, individuals with DS also start to experience loss of skills much earlier than the general population. In the next sections, we briefly review the evidence under each of the primary factors in our proposed framework as related to swallow function, speech production, and language/literacy in individuals with DS.

## Structural: oral-pharyngeal and neuroanatomical differences in individuals with DS

Normal swallowing depends on the rapid transfer of the prepared food or liquid bolus from the oral cavity to the stomach. To achieve manipulation, mastication, and containment in the oral cavity, coordinated movement between the lips, tongue, and jaw are crucial. As the bolus is transferred from the oral cavity posteriorly through the aerodigestive tract, adequate functioning of the soft palate, the larynx, and close coordination with the respiratory mechanism are important. Oropharyngeal anatomy in DS has distinctive features, even with expected intra-individual variations. These distinctive features are particularly relevant in the structures involved in swallowing. Common facial features include reduced mouth width and prominent lips, reduced size of hard palate, variety of dental anomalies, and relative macroglossia (Sforza et al., 2012). Compared to those without DS, individuals with DS have mid-face hypoplasia (Uong et al., 2001), a relatively small maxilla, but typical sized mandible (Allanson et al., 1993), dysmorphology of cranial base, maxilla, and mandible (Suri et al., 2010), and reduced palatal volume (Bhagyalakshmi et al., 2007; Dellavia et al., 2007). Additionally, airway abnormalities such as laryngomalacia, tracheomalacia, and bronchomalacia are frequent in individuals with DS (Bertrand et al., 2003).

Neuroanatomical differences may also play a role in the functioning of these complex tasks. Recent neuroanatomical studies, including MRI studies of adolescents with DS, have reported smaller cerebellar volumes, compared to age matched neurotypical peers, and other structural brain differences that are relevant to swallowing, speech, and language disorders (Wilson et al., 2019). Evidence suggests reduced volumes of total gray and white matter, cortical lobar, hippocampal, and cerebellar regions (Hamner et al., 2018). Individuals with DS may experience premature brain aging, with accelerated volume loss. The incidence of age-related cognitive decline and dementia is greater in adults with DS compared to the general population and develops earlier in life (Cole et al., 2017). In fact, older patients with DS show neuropathological changes characteristic of Alzheimer’s Dementia, including increased cerebral beta-amyloid deposits, neurofibrillary tau tangles, neuritis plaques, and neuron cell loss (Cole et al., 2017). Collectively, these anatomical changes could impact swallowing, speech, and language outcomes in individuals with DS.

## Sensation: sensory differences in individuals with DS

Sensory information allows individuals of all ages to internally perceive, recognize, and engage with their external environment (Bruni et al., 2010). Because each movement has a sensory consequence, traditional motor control theories highlight the tight temporal synchrony between sensory information and motor response needed for learning and maintaining skilled behaviors (Shumway-Cook and Woollacott, 2012). Thus, any alterations in sensation may impact motor plans and any alterations in structures could influence sensory feedback. One motor control theory that applies this concept for learning speech is the Direction into Velocity of Articulators (DIVA) model (Tourville and Guenther, 2011). The purpose of this computer-generated model of speech development is to highlight how children learn speech motor control through the interaction between auditory and somatosensory feedback from motor movements. Briefly, each time a child babbles, they receive auditory and somatosensory feedback that can be used to inform their motor plan. Each successive babble or speech attempt provides more information for the child to update their motor plan. As children learn, they continually update their motor plans through sensory feedback. Using the DIVA model as a basis, it is clear that oropharyngeal somatosensation, along with audition, is a crucial element for learning accurate speech production (Guenther et al., 2006; Golfinopoulos et al., 2010).

### Hearing

Approximately 38–78% of individuals with DS experience hearing loss (Intrapiromkul et al., 2012; Clark et al., 2017). Changes in auditory sensation can be conductive or sensorineural, but both may be linked to structural alterations. In several studies, conductive hearing loss was found to occur in 1/3rd of the study participants with DS, and typically secondary to chronic ear infection and stenosis of the external auditory canal (Park et al., 2012; Clark et al., 2017). Sensorineural hearing loss in this population is also seen, with computed tomography scans showing inner near abnormalities including stenosis of the cochlear nerve canal and internal auditory canal in 25% individuals with DS (Intrapiromkul et al., 2012; Nightengale et al., 2017). As we will describe, hearing loss may contribute to difficulties with speech perception and phonological processing, thus also contributing to difficulties in development of oral language and emergent literacy skills (Laws and Hall, 2014; Manickam et al., 2016).

### Vision

Visual acuity development in DS has been found to follow a different developmental trend than typical peers (Purpura et al., 2019). Structures with reported abnormalities include the lid, iris, lens, retina, and cornea (Krinsky-McHale et al., 2014). These structural changes result in the increased prevalence of nystagmus, strabismus, astigmatism, and significant refractive errors in individuals with DS (Krinsky-McHale et al., 2014). Across the lifespan, the incidence of visual impairments increases with age such that by 60 years of age, 85% of individuals with DS had visual impairment (Krinsky-McHale et al., 2014). The sensation of vision has a limited role in a biophysiological model of speech and swallowing; however, vision deficits can have implications for learning, particularly literacy learning, cognitive functioning, and adaptive behavior. Additionally, vision can play a role in priming a person for efficient swallowing, specifically through its role of stimulating saliva production needed for the oral preparatory phase of swallowing.

### Somatosensation

The somatosensory system transmits touch, pressure, and relative body position information from peripheral receptors centrally to the brain to inform movement responses. This system may be impaired in individuals with DS (de Knegt et al., 2015). Using quantitative sensory testing methods, de Knegt et al. (2015) assessed 188 adults with DS to determine their abilities to discriminate temperatures, sharp and dull pressure, and to detect touch on their forearm. A decreased ability to distinguish between sharp-dull pain was found to be associated with IQ level as measured on a standardized test. Lower sensitivity to pain may be the result of a smaller mediodorsal thalamic nucleus in DS, as this structure is important in transmitting sensory information to the prefrontal cortex (de Knegt et al., 2015). The loss of high-quality somatosensory feedback can interfere with the ability to learn and maintain accurate motor plans necessary for speech and swallowing. It is possible some deficits in behavior may not be the result of peripheral sensory appreciation, but in the processing and use of sensory inputs for accurate motor planning (Will et al., 2019).

### Sensory processing

Sensory processing is the continuous integration of information from the senses, movement, and muscle position by the nervous system which monitors an individual’s response, including over-or under-responsiveness, difficulties with stimuli discrimination, and challenges with proprioception and motor planning (Miller et al., 2007; Will et al., 2019). Difficulty with sensory processing is common across individuals with neurodevelopmental disabilities and linked to maladaptive outcomes (Baranek et al., 2018). Using Dunn Sensory profiles, (Dunn, 1999) sensory processing and visual organization abilities of 206 children with DS were studied by Wuang and Su (2011). About 41% of their sample was reported to have a “definite difference” in low registration, 40% a “definite difference” in low endurance/muscle tone, and 39% showed a “definite difference” in sensory sensitivity (Wuang and Su, 2011). In a second study, almost half of children with DS experienced a definite difference in the low energy/weak, under-responsive/seeks sensation, and the auditory filtering domains (Bruni et al., 2010). The combined results of these studies point to differences in the way children with DS identify, process, and respond to various types of sensory information. These issues that arise in childhood might be expected not just to continue, but potentially to be magnified across the lifespan (Grieco et al., 2015).

## Individual and environmental factors

In addition to the above factors, several individual and environmental factors can impact functional behaviors. Some of these individual factors include cognition (Anil et al., 2019), dietary requirements for nutrition and medical needs (Wallace, 2007), food preferences (Field et al., 2003; Anil et al., 2019) etc. Although intellectual disability is a characteristic of DS (and thus could be considered within the “DS phenotype” as well), we have chosen to consider it an “individual” factor instead. In part, this reflects the broad spectrum of intellectual and adaptive functioning found in individuals with DS (Mégarbané et al., 2013; Carr, 1988). Additionally, a thorough evaluation of the impact of some of the characteristic cognitive features (related to attention, memory, etc.) would require a dedicated article of its own, beyond the scope of the current article.

Environmental factors such as parental anxiety and grief surrounding a DS diagnosis (Carr, 1988), cultural expectations, and access to care can impact speech, swallowing, and literacy outcomes (McCabe et al., 2011; McGrath et al., 2011; van den Driessen Mareeuw et al., 2020). Although discussing all these factors is beyond the scope of this paper, in the following section we include a discussion on the importance of cognition on speech, swallowing, language and literacy.

## Functional implications for swallow, speech, and language/literacy outcomes

In this section, we explore the potential interrelations between the information reviewed in the previous section and the functional outcomes of swallow, speech, language, and literacy. We first begin by considering the potential relationships at a broad/general level. We then offer two detailed examples of how phenotypic characteristics of DS could specifically affect each of the four functional outcomes of interest, as a model for how the other information in the upcoming section might play out across the functional domains. Not all of the direct relationships have been studied and warrant direct research.

## Functional implications for swallowing

While seemingly effortless for most adults, the production of a safe swallow and intelligible speech involves rapid and complex coordination of oral-motor structures and functions. This coordination: (a) relies on high-quality sensory feedback from the lips, tongue, jaw, and pharynx; (b) requires skilled, coordinated motoric control, and (c) is informed by and dependent on cognitive, linguistic, and perceptual skills for planning and execution.

Extensive research has documented significant problems with swallowing in both children and in adults with DS. For example, dysphagia has a higher documented prevalence in adults with DS relative to controls (Capone et al., 2020; Chicoine et al., 2021), and adults with DS are substantially more likely to die from choking than those without DS (Landes et al., 2020). Feeding/swallowing difficulties are likely common in individuals with genetic conditions due to the complex interaction between medical, anatomical, physiological, and behavioral factors. In a study by Anil et al. (2019), the parents of 17 children with DS and 47 typically developing children completed a questionnaire regarding feeding. The most prevalent feeding problems in the oral phase were increased oral hold, increased duration for bolus manipulation, difficulty chewing, and inappropriate oral transit (Anil et al., 2019). In the pharyngeal phase, delayed posterior transit and aspiration were reported. In the esophageal phase, the researchers postulated that reduced muscle tone may result in increased vomiting, poor digestion, and gastroesophageal reflux (Anil et al., 2019). Additionally, considering sensory information that’s important to swallowing, changes to taste and smell have been identified in individuals with DS across the lifespan, possibly related to structural differences that may impact nasal health, resulting in hypoplasia (Chen et al., 2006). Taste and smell deviations can impact swallowing because they are an important sensory input element for the motor output of an efficient swallow.

Oral-motor skills can also be impacted, with possible weak lip closure, compression pattern without the use of intraoral suction, tongue thrusting, and chewing difficulties (Cooper-Brown et al., 2008; Anil et al., 2019; Ross et al., 2019). In a retrospective chart review of 158 children with DS, oral motor difficulties occurred in 63.8% and oral sensory difficulties in 20.3% of the sample (Jackson et al., 2016). Oral sensory difficulties included both oral hyposensitivity and oral hypersensitivity. Ross et al. (2019) found that many children with DS only ate “easy” low-textured food and refused to chew. This downstream effect of oral sensory changes and textural preference can result in a lack of diversity in dietary and nutritional intake (Ross et al., 2019).

In the pharyngeal phase of swallowing, studies report frequent “silent” aspiration and what was characterized as deep laryngeal penetration in most or all participants (Frazier and Friedman, 1996; Jackson et al., 2016). Evidence suggests aspiration may be related to hypotonia of the pharyngeal musculature in infants, perhaps suggesting a generalized hypotonicity in individuals with DS (Shott, 2006). The lack of a cough response to aspirated materials indicates decreased laryngeal sensation.

In addition to dysphagia, individuals of all ages with DS can present with chronic pulmonary problems and obstructive sleep apnea, that contribute to respiratory problems like recurrent pneumonias, recurrent upper and lower respiratory tract infections, and even respiratory failure (Bertrand et al., 2003). Feeding and swallowing difficulties thus become more significant due to an increased risk of aspiration, lower immune system response, and possible support needs for activities of daily care and living. In fact, respiratory illness is one of the most common causes of mortality in DS (Landes et al., 2020). Aspiration from food and liquid ingestion is as a top cause for respiratory illness and mortality in children and adults with DS (Frazier and Friedman, 1996; Landes et al., 2020). Future studies should analyze whether comorbidities that are associated with DS are prognostic of the presence, severity, and longevity of dysphagia.

Finally, the act of swallowing requires coordination between physiologic and sensorimotor responses, visual recognition of food, motor planning, wish to eat, and essentially, cognitive awareness (Rogus-Pulia et al., 2015). Particularly in the oral phase of swallowing, cognitive deficits in attention, decision making, recognition and orientation can impair swallowing (Langmore et al., 2007). Because decreased attention and impulsivity are frequently reported in individuals with DS (Capone et al., 2006), they may be at risk for increased difficulties in certain aspects of swallowing, although this possibility requires direct study. Additionally, the brain volume loss and premature aging experienced by individuals with DS may lead to the need to adapt these deficits to a constantly evolving mechanism, potentially creating new and lifelong difficulties with complex oral motor behaviors.

## Functional implications for speech, language, and literacy outcomes

The combination of structural anomalies, peripheral sensory changes, alterations in sensory processing and individual factors such as cognition likely impact speech behavior. Across the lifespan, individuals with DS experience difficulties with intelligible speech that impact vocational social, independent living, and self-advocacy outcomes, among others (Kumin, 1994; Fawcett and Peralego, 2009; Kent and Vorperian, 2013). Children developing typically usually reach 100% intelligible speech by 4 years of age however, it is unusual for children with DS to reach 100% speech intelligibility at that age (Kumin, 2006). Indeed, Martin et al. (2009) note that “nearly all individuals with DS may be difficult to understand at least some of the time” (p. 115). Hearing loss and auditory discrimination difficulties make it more difficult for children with DS to perceive the subtle differences between sounds, which again may contribute to the difficulty in producing speech sounds (Kumin, 2006) as well as learning foundational literacy skills such as phoneme-grapheme correspondence.

Physiologic findings suggest that speech and voice problems such as dysarthria, apraxia, voice and resonance problems may be associated with features such as limited tongue moment during vowel production which results in reduction in acoustic vowel space, articulatory working space, and articulatory speed (Wilson et al., 2019). Other factors that are associated with their speech and voice disorders include craniofacial and laryngeal dysmorphologies, motor impairments, phonological delay or disorder, dysfluency, and hearing loss (Rosin et al., 1988; Kent and Vorperian, 2013; Wong et al., 2015).

Little direct research has examined possible relations between speech production, cognition, and language and literacy outcomes. However, Cleland et al. (2009) evaluated whether global measures of language and cognitive functioning correlated with overall intelligibility in 15 youth with DS; they found little correlation. However, we would argue language-speech relationships are not global (as measured by Cleland et al., 2009), but rather represent more specific relationships between speech production and cognitive and linguistic demands (such as working memory and/or syntactic complexity, respectively). For instance, expressive grammar is a particular challenge in DS (Rvachew and Folden, 2018; Abbeduto and McFadd, 2021). Studies of children with typical development and those with language impairments have demonstrated a “trade-off” between speech and language, such that when linguistic demands increase, speech movement becomes more variable and phonemic accuracy decreases (Masterson and Kamhi, 1992; Maner et al., 2000; Seeff-Gabriel et al., 2010). Given this speech-language tradeoff in other populations, and the selective difficulty in grammar in DS, it is possible that when demands of either speech or of language increase, there is a toll on the other. An individual seeking to produce a particularly difficult spoken token (“crocodile”) may sacrifice syntactic complexity, producing it either in the context of less-complex syntax and/or making syntactic errors.

Sensation limitations also have implications for language and/or literacy outcomes. Reduced access to the speech signal likely affects speech perception as well as speech production. Difficulties with speech perception or processing in turn affects language comprehension, at least for spoken input. For instance, a child who cannot distinguish between minimal phonetic pairs (“bat”/“pat”) may in turn have difficulty producing them correctly in their speech, and with mapping the words to their respective semantic concepts linguistically and with acquiring the phoneme/grapheme linkages needed for literacy (e.g., see Abbeduto et al., 2007, for a discussion of the role of auditory and phonological processing on language and literacy outcomes in individuals with Down syndrome). A child who is not perceiving final sounds, such as/t/or/d/, will in turn be challenged in incorporating those into expressive or receptive syntax, as many morphemes occupy that final position. Limitations in vision will compound the difficulties with phoneme-grapheme acquisition as well as other literacy outcomes (whole word reading, decoding; Woodhouse, 2005). Finally, limitations in oral somatosensation can result in difficulty identifying where the tongue is in relation to the palate or teeth, resulting in the speech production challenges that, as noted earlier, might in turn compromise production of complex expressive language (in particular, syntax).

## Two examples illustrating the value of an integrated biophysiological approach

**FIGURE 2:**
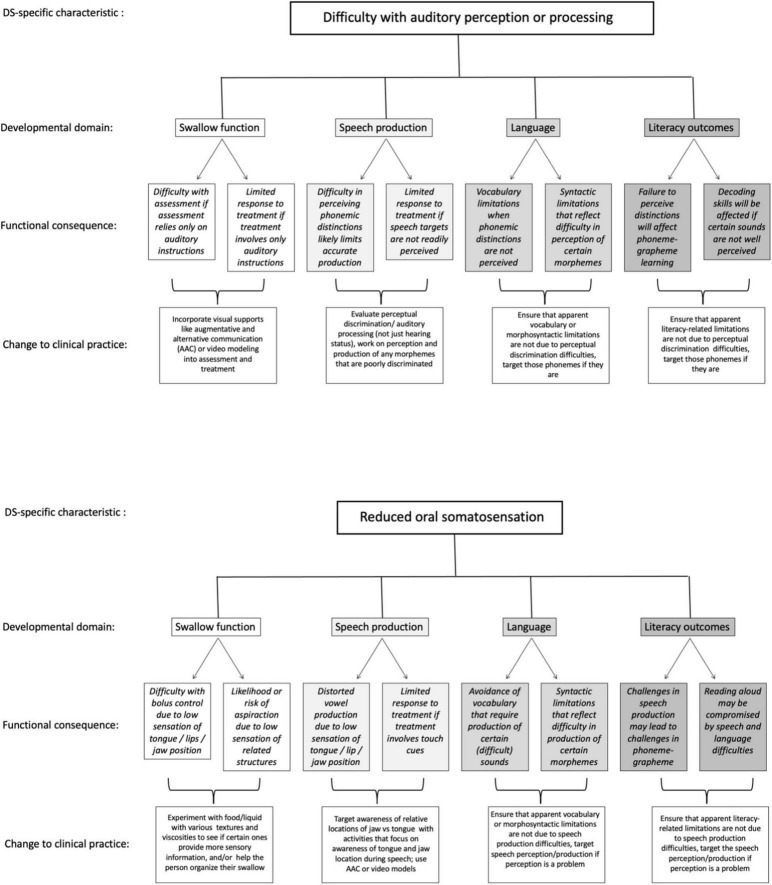
Two examples of specific ways that phenotypically linked characteristics can affect each of the four domains of swallow, speech, language, and literacy in DS, and implications for clinical practice.

To this point, we have described at a general level some of the implications of structural and functional characteristics of DS for swallow, speech, language, and literacy outcomes. We now briefly offer specific examples of how two of the phenotypic characteristics in DS might impact each one of the four domains of function, and offer examples of the potential clinical implications for service provision. The examples are summarized in Figure 2 and described in the text, and illustrate the potential value of our proposed integrated approach. Some of the implications and suggestions in the figure involve reflection on language (metalinguistics, and some metacognitive skills). Such reflection may not be within the repertoire of all individuals with DS, but certainly will be within the repertoires of many of them, given that metalinguistic skills emerge at developmental age 5–7 years (Bialystok, 1986). Moreover, recent research has illustrated that when given appropriate instruction and targeted input, individuals with DS can learn and engage in metacognitive or abstract/higher order cognitive reflection (Engevik et al., 2016; O’Neill and Gutman, 2020). The framework therefore offers suggestions for targeted interventions even if individuals do not currently appear to be reflecting on their own speech.

Panel A of Figure 2 presents examples of how difficulty in distinguishing phonemic distinctions (i.e., perceiving subtle differences in speech input) might impact each of the four functional domains. In swallowing, assessment or interventions that rely solely on auditory instructions/input might have limited value if individuals have difficulty perceiving distinctions in speech input. Consequently, visual communication supports that augment spoken input might be critically important to ensure accurate understanding of instructions for assessment or intervention in swallowing (see, for instance, Santoro, 2022). For speech, if an individual cannot discriminate between phonemes in input, they are unlikely to clearly produce those distinctions in their own spoken output, resulting in lower speech intelligibility. For language outcomes, vocabulary might be affected if individuals avoid or do not understand vocabulary words with certain phonemic distinctions; syntax might be similarly affected if phonemic distinctions that signal different morphemes are not well perceived in spoken input. Similarly, difficulty perceiving distinctions in speech input will likely interfere with literacy outcomes such as phoneme-grapheme learning, where phonemic input is matched to the graphemic representation, and/or decoding skills. Implications for service provision include making sure we assess not only hearing status, but also more specifically perceptual discrimination of speech sounds. This information can help to target services to support perceptual discrimination and to highlight instances in which limitations in vocabulary, syntax, or literacy outcomes relate not just to linguistic or cognitive challenges but may also reflect difficulty in perceiving important distinctions in spoken input.

Panel B of Figure 2 lays out the implications for a very different phenotypic characteristic, that is, potential low oral somatosensation, on each of the four domains. In swallow, individuals who are less attuned to the coordination of their lip, tongue, and jaw will likely be less able to identify the necessary orofacial postures needed to achieve accurate or efficient functional swallowing behaviors, including controlling the bolus within the oral cavity and minimizing aspiration of food or liquid. In that event, it might be necessary to explore various food textures or liquid types (e.g., carbonated or thickened liquids), that might enhance an individual’s ability to sense the food or liquid and better control swallow functioning. In speech, reduced tongue somatosensation likely influences low vowel production, due to less contact with molars compared to high vowels, resulting in lower speech intelligibility (Gick et al., 2017). Speech interventions that rely on touch cues (e.g., Hoose, 2019) will likely be less effective if individuals cannot sense the intended cues. In that event, service provision might target awareness of relative locations of jaw vs. tongue, using activities that focus on awareness of tongue and jaw location during speech, and use AAC or video modeling in that process. In language and literacy, if certain sounds are more difficult to produce due to somatosensory limitations, both vocabulary and syntax may be compromised due to avoidance of words or morphemes containing those sounds, and these same challenges may lead to difficulty with phoneme-grapheme acquisition as well as oral reading (reading aloud). Simply understanding that vocabulary, syntax, or literacy challenges may relate to physical difficulty with certain speech sounds will help to target interventions that include a focus on somatosensation.

## Conclusion and applications to other clinical populations

A biophysiological model that combines structure, sensation, and individual factors for oropharyngeal motor activities provides an integrated approach for in-depth assessment and treatment of speech, swallowing, language, and literacy in individuals with DS. A thorough understanding of these factors and how they impact functional outcomes can be used to construct better, individualized treatment plans for individuals with DS. If one area of the model is identified as challenging to an individual, another factor within the model could be used to compensate. For example, to help achieve accurate motor movements, enhanced sensory cues for correct placement could be provided. If the individual has difficulty recognizing or processing sensory information, the therapist might increase inputs through another channel or provide feedback through multiple sensory modalities. In swallowing this can be achieved with the use of foods of various textures, stronger tastes, or even carbonation. In language and literacy, this can be achieved through multi-modal input that includes both auditory but also visual supports (see, e.g., Wilkinson and Finestack, 2021).

Although individuals with a common etiology may share similar structural and sensory changes, individual factors are important to consider, as they will vary widely. Individual factors in the assessment and treatment of swallowing could include food preferences, dietary restrictions, and nutritional needs to maintain overall health. For speech production and accuracy, individual factors could include the context in which the individual is communicating or language abilities. While not discussed in detail in this paper, the authors would like to emphasize the importance of considering these factors thoroughly while serving individuals with DS.

The authors were constrained in the amount of detail that could be provided, due to page limitations. However, many of the proposals in this article have not yet received direct research attention, and it is our hope that the outline we have provided will encourage future research on potential interrelationships. As it stands, this multidimensional, biophysiological approach to understanding complex, skilled behavior forms the basis for clinical interventions and has multiple functional implications. The purpose of this paper was to demonstrate the utilization of an adapted biophysiological framework to consider multiple dimensions that influence performance of skilled oral motor behaviors. DS was used as a clinical example to enumerate the use of the framework. However, the idea of interrelated factors in a multidimensional framework can be used with any clinical population, highlighting aspects that influence behavior in each population. For example, increased sensory processing difficulties in individuals with autism or specific neuroanatomical differences in individuals with cerebral palsy. Utilizing the framework in this way can assist in completing a holistic clinical evaluation that would aid in targeted treatment planning.

## Data availability statement

The original contributions presented in this study are included in the article/supplementary material, further inquiries can be directed to the corresponding author.

## Author contributions

AM and NE conceived and designed the framework presented in this study, organization, and drafting of the manuscript. LL contributed to the organization and drafting. KW provided input in conceptualizing, senior authorship, applications to DS, and drafting. All authors contributed to the article and approved the submitted version.
